# Pericentric Inversion of Chromosome 9 in Twins With Cyclopia: A Rare Entity

**DOI:** 10.7759/cureus.34562

**Published:** 2023-02-02

**Authors:** Nik Ahmad Zuky Nik Lah, Fahisham Taib, Erinna Mohamad Zon, Engku Husna Engku Ismail, Aziati Azwari Annuar

**Affiliations:** 1 Department of Obstetrics and Gynecology, School of Medical Sciences, Universiti Sains Malaysia, Kubang Kerian, Kota Bharu, MYS; 2 Department of Pediatrics, School of Medical Sciences, University Sains Malaysia, Kubang Kerian, Kota Bharu, MYS; 3 Department of Obstetrics and Gynaecology, School of Medical Sciences, Universiti Sains Malaysia, Kubang Kerian, Kota Bharu, MYS; 4 Department of Obstetrics & Gynaecology, School of Medical Sciences, Universiti Sains Malaysia, Kubang Kerian, Kota Bharu, MYS; 5 Human Genome Centre, School of Medical Sciences, Universiti Sains Malaysia, Kubang Kerian, Kota Bharu, MYS

**Keywords:** cyclopia, alobar holoprosencephaly, patau syndrome, dichorionic diamniotic twins, pericentric inversion of chromosome 9

## Abstract

Pericentric inversion of chromosome 9 (inv(9)) is one of the most common variants seen in a normal human karyotype that occurs during meiosis.

Despite being categorized as a normal variant, some studies using classical cytogenetics have recently shown that inv(9) could be associated with azoospermia, congenital anomalies, growth retardation, and rarely with abnormal karyotype. However, there is no reported association with cyclopia. Interestingly this genetic variant involves twin fetuses.

A 36-year-old multiparous lady with dichorionic diamniotic twin pregnancy presented to the fetomaternal unit with fetal growth restriction at 34 weeks of gestation. An ultrasound scan revealed both have microcephaly, fisting hands, holoprosencephaly, and proboscis suspicious of Patau syndrome. Amniocentesis was not issued due to late pregnancy and guarded prognosis. The mother presented with pre-eclampsia at 35 weeks of gestation. The pregnancy managed to prolong up to 36 weeks after which caesarean section was performed due to the leading twin being in a transverse lie. Two baby twin girls were born 3 minutes apart with microcephaly and cyclops appearance. Chromosomal analysis of both twins revealed similar karyotypes of 46, XX, inv(9)(p11,q13).

Pericentric inversion of chromosome 9 is regarded as a normal chromosomal variation in the general population, but in twins with cyclops is considered rare. Early referral to a tertiary hospital for twin management is highly required. It may identify fetuses with such abnormalities and counsel the parents with appropriate management.

## Introduction

The pericentric inversion 9 (inv(9)) variants, inv(9)(p11q13) or inv(9)(p12q13), are considered the most prevalent chromosomal variations detected perinatally [1]. The prevalence of inv(9) in the New York City population based amniotic fluid trypsin Giemsa banding (GTG) for prenatal diagnosis was 3.57% in the African-American population, 2.42% in Hispanics, 0.73% in Whites, and 0.26% in Asians [1]. Inv(9) was noted in 1.7% -1.9% of the total newborn cases [2,3]. The incidence of inv(9) was significantly higher in female fetuses than males in prenatal diagnosis and in a population study [4,5].

In general, pericentric inversion entails a rearrangement of chromosome material at its centromere during meiosis that results in gametes with an uneven distribution of its materials [6]. The gametes either have a duplication involving the distal segment of the short arm (p-arm) of the chromosome and a deletion of the distal segment of the long arm (q-arm) of the chromosome or conversely [6]. This subsequently brings about miscarriages, non-viable embryos, or various anomalies [6]. Inv(9)(p11q13) was reported to have various congenital fetal anomalies, including, polydactyly, club foot, small bowel malrotation, pulmonary stenosis, cardiomyopathy, arrhythmia, and intrauterine growth restriction [2]. However, Jeong et al. concluded that the inv(9)(p11q13) rate in patients with congenital fetal malformations was similar to the ordinary population [2].

Cyclopia is a rare congenital anomaly identified by the presence of single fused eyes located in the centre of the face. In contrast, the fusion of two eyes in varying degrees is known as synophthalmia [7]. Both result from a failure of the embryonic prosencephalon to appropriately divide the orbits of the eye into two cavities [8]. This is a part of alobar holoprosencephaly (HPE) in which there is total or near total interhemispheric separation, absence of interhemispheric fissure and falx cerebri with single midline forebrain ventricle, and a few other brain anomalies [8]. It is easily recognized prenatally by ultrasound with a single brain ventricle, microcephaly, cyclopia, proboscis, and facial cleft [9].

The cyclopia prevalence is 1.0 per 100,000 births [10]. It has a heterogeneous etiology, which could result from chromosomal defects, genetic mutations, or environmental teratogenic factors [8]. It has been reported as between 10% and 18% of the HPE [11]. Trisomy 13 is the most frequent chromosomal disorder associated with HPE [12].

## Case presentation

A 36-year-old lady gravida 9, para 4+4 (abortions) at 34 weeks of gestation, was referred to our fetomaternal unit for twin pregnancy with fetal growth restriction. An early scan confirmed that she had dichorionic diamniotic (DCDA) twinning. Both parents were first cousins and had four previous histories of abortions with one deceased term baby identified clinically as Patau syndrome previously. There was no reported cyclopia or inv(9) previously in the family.

**Figure 1:**
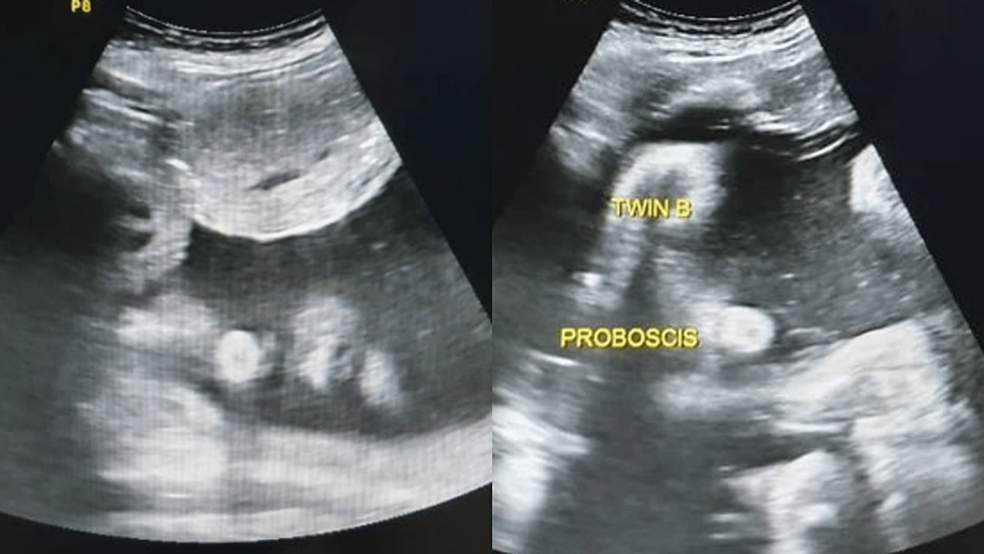
Ultrasound images of the twins at 35 weeks. The first twin is on the left, both twins show proboscis

**Figure 2:**
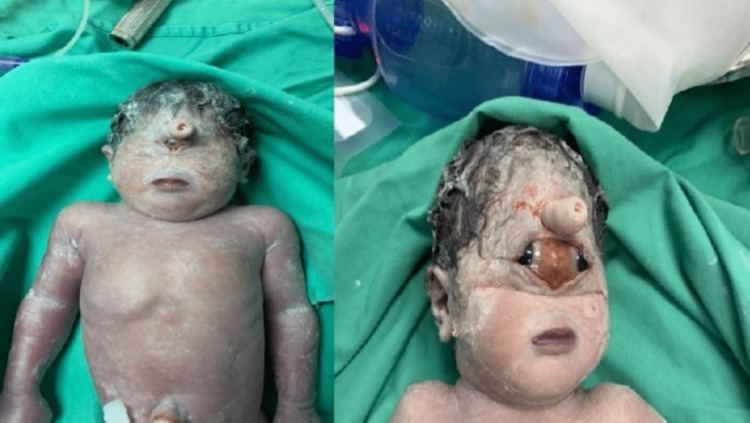
The first twin (left) had a true cyclopia, and the second twin (right) had synophthalmos features

She was found to have polyhydramnios and intrauterine growth retardation twins. Ultrasound scan showed a twin with microcephaly, fisting hands, holoprosencephaly, and proboscis (Figure 1). Parents were informed of a guarded prognosis, and amniocentesis was not performed in view of late pregnancy. Parents were counseled about the poor outcome and aggressive resuscitation of the twin is unlikely to help the twin survive. The mother presented with pre-eclampsia at 35 weeks of gestation and the scan showed fetuses in the transverse lie position, leading to a caesarean section at 36 weeks. Two baby twin girls were born 3 minutes apart with microcephaly, cyclops appearance, and proboscis (Figure 2). No cleft lip or cleft palate was noted. All other organs of the babies were normal. The Apgar for both babies were 3 at 1 minute and 2 at 10 minutes. The weights were 1.46kg and 1.68kg, respectively. Active resuscitation was not attempted as the condition was incompatible with life. Babies passed on approximately 25 mins after delivery with the presence of the father around them. Cytogenetic analysis of both twins revealed similar karyotypes of 46, XX, inv(9)(p11,q13).

## Discussion

Twins have been linked with paternal chromosomal inversion, inv(9)(p11,q13) [13]. Orioli et al. reported that only 6 of 231 infants with cyclopia were twins in 25.6 million births [8]. Kallen et al. reported seven pairs among 103 infants with cyclopia in ten million births [10]. In this case, there was a dichorionic diamniotic twinning, and the first twin had a true cyclopia while the second twin had synophthalmos features. Both are females and with proboscis.

Cytogenetic analysis of both twins revealed similar karyotypes of 46, XX, inv(9)(p11,q13), considered to be a normal population variant revealing a pericentric inversion on one of the chromosomes 9 in which breakage and reunion have occurred at bands 9p11 and 9q13. However, their zygosity could not be determined as the test was not initially requested. Inv(9)(p11q13) has been reported with various congenital abnormalities but not cyclopia or synophthalmia [8]. We cannot comment on inherited structural rearrangements in the twins as no chromosomal analysis was carried out on both parents. They were not keen for their blood to be tested. However, a study by Jeong et al. revealed inv(9)(p11q13) chromosomal aberrations with congenital abnormalities were de novo heterozygous in nature [2]. A study by Merrion and Maisenbacher concluded that the inversion 9 variant in the parents is not linked with an increased risk for uneven chromosome products or aneuploidy incidences [6].

The exact causes of cyclopia are still largely unknown. Embryologically, there were two sets of lens fibers in the cyclopia or the synophthalmia, and it suggested that the retinal field might have been divided incompletely during its early development which brought about the fusion of the two primordia to form a single eye [7]. This eye development embraces an exquisite interplay between various genes and transcription factors; perhaps the most paramount gene involved is sonic hedgehog factor (Shh). Shh expressed from the prechordal plate leads to down-regulation of the PAX6 gene and activation of the PAX2 gene, which gives rise to the single eye splitting into two. Shh stimulates cell proliferation, tissue patterning, and differentiation at multiple points during the separation of visual fields in the neural plate, which leads to two separate eyes and orbital cavities [14].

Defects in the Shh protein or the Shh signaling pathway leads to holoprosencephaly [15]. Shh is also required for the brain to develop into discrete left and right lobes. If a single forebrain defect is present (alobar holoprosencephaly), a single eye will likely develop. Lack of down-regulation of PAX6 and lack of expression of PAX2 due to a defect of Shh function can lead to the above conditions [15].

Most babies with cyclopia are either stillborn or pass away shortly after birth because their brain and other organs are abnormal and incompatible with life.

## Conclusions

Pericentric inversion of chromosome 9 is regarded as a normal chromosomal variation in the general population. However, in twins with cyclops, it is considered rare. Early referral to a tertiary hospital for twin management is highly required. It may identify fetuses with such abnormalities and counsel the parents with appropriate management.
